# Parental compliance - an emerging problem in Liverpool community child health surveys 1991-2006

**DOI:** 10.1186/1471-2288-12-53

**Published:** 2012-04-20

**Authors:** Gibby Koshy, Bernard J Brabin

**Affiliations:** 1 Child and Reproductive Health Group, Liverpool School of Tropical Medicine, Pembroke place, Liverpool, L3 5QA, UK; 2 Department of Community Child Health, Royal Liverpool Children’s Hospital, AlderHey, NHS Trust, Liverpool, UK; 3 Emma Kinderziekenhuis, Academic Medical Centre, University of Amsterdam, Amsterdam, Netherlands

**Keywords:** Compliance, Cross-sectional, Survey

## Abstract

**Background:**

Compliance is a critical issue for parental questionnaires in school based epidemiological surveys and high compliance is difficult to achieve. The objective of this study was to determine trends and factors associated with parental questionnaire compliance during respiratory health surveys of school children in Merseyside between 1991 and 2006.

**Methods:**

Four cross-sectional respiratory health surveys employing a core questionnaire and methodology were conducted in 1991, 1993, 1998 and 2006 among 5-11 year old children in the same 10 schools in Bootle and 5 schools in Wallasey, Merseyside. Parental compliance fell sequentially in consecutive surveys. This analysis aimed to determine the association of questionnaire compliance with variation in response rates to specific questions across surveys, and the demographic profiles for parents of children attending participant schools.

**Results:**

Parental questionnaire compliance was 92% (1872/2035) in 1991, 87.4% (3746/4288) in 1993, 78.1% (1964/2514) in 1998 and 30.3% (1074/3540) in 2006. The trend to lower compliance in later surveys was consistent across all surveyed schools. Townsend score estimations of socio-economic status did not differ between schools with high or low questionnaire compliance and were comparable across the four surveys with only small differences between responders and non-responders to specific core questions. Respiratory symptom questions were mostly well answered with fewer than 15% of non-responders across all surveys. There were significant differences between mean child age, maternal and paternal smoking prevalence, and maternal employment between the four surveys (all p<0.01). Out-migration did not differ between surveys (p=0.256) with three quarters of parents resident for at least 3 years in the survey areas.

**Conclusion:**

Methodological differences or changes in socio-economic status of respondents between surveys were unlikely to explain compliance differences. Changes in maternal employment patterns may have been contributory. This analysis demonstrates a major shift in community parental questionnaire compliance over a 15 year period to 2006. Parental questionnaire compliance must be factored into survey designs and methodologies.

## Background

Compliance is critical for representativeness for school based epidemiological surveys and it is becoming increasingly difficult to achieve high compliance in these surveys in urban areas in industrialised settings. Several sequential school based surveys were carried out in Merseyside between 1991 and 2006. This provided an opportunity to assess participant questionnaire compliance in the same school populations over a prolonged period. In the early 1990’s the dock area of Liverpool had substantial coal dust pollution following importation of steam coal from South America. The first Respiratory Health Survey was completed in 1991, amongst school children aged 5-11 years and achieved a parental compliance for questionnaire completion of 92%. Analysis of this data showed a spatial association of childhood asthma symptoms which was associated with the concentrations of airborne dust pollution on contour maps [1,2]. As a consequence a total of three further cross-sectional surveys were undertaken in the same primary schools in 1993, 1998, and 2006, using essentially the same parental questionnaire and survey methodology [3]. These studies had focussed on childhood health outcomes in relation to pregnancy smoking, residential location, socio-economic status and factors related to asthma, obesity and Attention Deficit Hyperactivity Disorder (ADHD) [4-14].

The surveys provide a unique opportunity to assess parental participant compliance in the same survey schools over a 15 year period. Decline in response rates to general population surveys have been demonstrated over recent decades [15], and with even steeper declines seen over recent years [16,17,18,19]. Insight into participant characteristics which modify such study participation is essential in order to facilitate improving questionnaire response rates, as well as for establishing the validity of survey findings.

Parental compliance fell sequentially in the consecutive Merseyside surveys over the fifteen year period, and the present analysis aimed to describe how response rates to specific questions varied over time and how they related to socioeconomic status and demographic characteristics of parents of children attending these primary schools.

## Methods

### Study population

**Figure 1:**
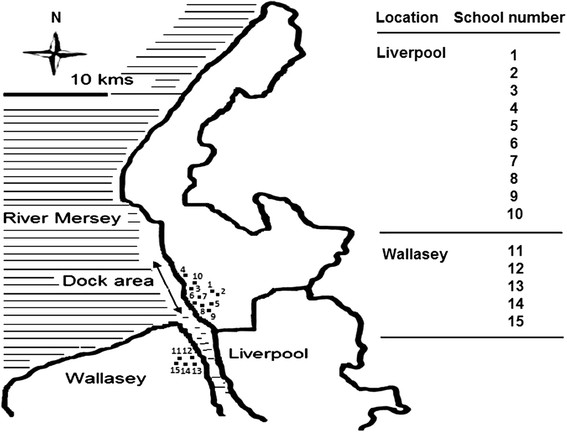
Geographical location of primary schools in Liverpool and Wallasey surveys.

Four cross-sectional respiratory health surveys were completed in 1991 (n=1872), 1993 (n=3746), 1998 (n=1964) and 2006 (n=1074) among primary school children (5-11 years). The four surveys were conducted in the same 15 schools, except for 1998, when 10 of the originally surveyed schools were included. The survey period was September to December for all surveys. The total number of questionnaires distributed was 2035 (15 schools; alternate children) for 1991, 4288 (15 schools; all children) for 1993, 2514 (10 schools; all children) for 1998 and 3540 (15 schools; all children) for 2006. The schools the children attended were within the same geographical areas of South Sefton in Liverpool (10 schools) and Seacombe in Wallasey (5 schools) (Figure 1). The schools selection was based on location in the original dust-exposed area in Liverpool (10 schools), with an additional 5 schools located upwind from the exposed area and south of the river Mersey (Wallasey). A smaller sampling frame (10 instead of 15 schools) was used in 1998 due to substantially decreased community concern over airborne dust pollution in the study area. The 5 Wallasey schools originally included in the 1991 and 1993 surveys were located outside the known dispersion areas for the coal dust which had lead to the original community concerns about airborne pollution. These locations were all lower socio- economic areas and had comparable Townsend Deprivation Indices based on postcode classification [1,4].

### Merseyside Respiratory Health Surveys

The Merseyside Respiratory Health Surveys were first undertaken in 1991 because of the effects of airborne dust pollution on asthma risk among children attending primary schools and living around the Sefton Dock area [1,4]. The Liverpool docks were well known as a route for importation of a range of commodities and steam importation and handling of coal started on a large scale during the period from 1980 to 1990. These dock activities resulted in air pollution from black dust which affected people living in and around the dock area. Children attending primary schools adjacent to the Bootle dock area of Merseyside were first reported by school teachers in 1990 and 1991 to be experiencing frequent asthma episodes which required inhaler use. To investigate these complaints and determine the prevalence of respiratory morbidity in primary school children in the area exposed to airborne dust, a Steering Committee was established under the auspices of Sefton Health Authority. This lead to the completion of a Respiratory Health Survey in 1991, in school children aged 5-11 years. The main objective was to determine whether the school children in a specific locality, exposed to airborne coal dust pollution had an excess of respiratory symptoms compared to school children of the same age from non - dust exposed areas of Merseyside of comparable socio-economic status (Wallasey). A link with airborne dust pollution was demonstrated [2,16] and as a consequence further sequential surveys were planned in the same primary schools to assess the health trends in children, and to monitor the effects of proposed environmental interventions through community surveillance. As a consequence of these activities, the changes in parent-reported childhood asthma prevalence in Merseyside have been assessed in consecutive cross-sectional surveys undertaken in 1991, 1993, 1998 and 2006. The 1993 survey aimed to determine environmental and host risk factors for respiratory symptoms and to monitor asthma over a two year period [1,2]. The 1998 survey involved on-going monitoring of respiratory symptom prevalence [3,7,14]. The 2006 survey was conducted to establish if the increasing prevalence of childhood asthma had plateaued, as well as to determine the prevalence of obesity and other child health outcomes in relation to pregnancy smoking [3,5-7,11,13,14].

Children (5-11 years) attending these schools were invited to take part in the study following a letter to parents requesting written informed consent. Parents were required to complete a questionnaire and give permission to measure their child’s height and weight. The sampling frame for each of the four surveys was:-1991(alternate children in school class registers) and for 1993, 1998 and 2006 (all children on the school class registers). In 2006 one primary school surveyed between 1991 and 1998 had since closed and this was replaced with a school in the same geographical locality as the school which had closed (less than 0.5Km). Schools comprised almost wholly Caucasian pupil populations and this composition had changed little during the survey periods [17].

### Parental questionnaire and data collection procedure

Parents received questionnaires through their child’s school teacher with an information letter and written consent form, and a supporting letter from the school’s headmaster. Class registers were used to identify children. Forms were taken home by individual children and teachers requested children to return envelopes containing the questionnaire within one week.

The original questionnaire was a modified version of one designed and used by Clifford et al [18]. The questionnaire was further modified in 1998 and 2006 to include core questions related to the ISAAC protocol (International Study of Asthma and Allergy in Childhood) [19] which ensured comparable diagnostic information on the epidemiology of asthma and across surveys.

For doctor diagnosed asthma status the question asked was ‘Has your child ever been diagnosed by a doctor as having asthma or bronchial asthma?’ Information regarding birth weight, maternal risk factors including smoking history during pregnancy, and respiratory problems were collected. The same questionnaire was used and the core questions were similar for all surveys with slight modifications in the 1998 and 2006 surveys, in which additional questions on parental smoking practices and maternal alcohol consumption were included. Envelopes with questionnaires for absent children were forwarded to parents, if the child re-attended within the period of each school survey (two weeks). Class lists were made available by head teachers to enable compliance to be calculated. A single reminder letter was sent for unreturned questionnaires.

For anthropometric measurements, children were asked to remove their shoes and heavy garments. Height was measured to the nearest millimetre using a Minimeter scale, which was placed against a vertical surface in the school building. Height was measured with the child standing erect, and measured by the same observer for all children in a particular survey. Weight was measured to the nearest 100 grams using electronic scales (Seca). These were pre-calibrated and the weight reading checked prior to each survey.

Data for the 1991, 1993 and 1998 surveys were screened for outliers, cross-checked with the original questionnaires, and non-biological values were omitted. Data was manually entered for the 1991, 1993, and 1998 surveys, and optically read with manual checking for the 2006 survey. Optical reading of questionnaires was completed using the Read Soft software program. Once data was ready in word pad form it was directly transferred to Microsoft excel, and then to SPSS (Statistical Package for the Social Sciences) for analysis.

It was not possible to validate parental responses to health parameters with general practice medical records.

### Data analysis and statistical methods

The analysis assumes that the population is representative of a single Caucasian ethnic group and that the denominator school populations represented the same geographic catchment areas for the 15 year survey period. The primary hypothesis examined whether question compliance was related to changes in socio-economic status.

Statistical analysis was performed using the Epi-Info, and SPSS (version 17) and all data was anonymised. Distributions for demographic, social and respiratory characteristics were compared using Chi-square, for comparison of proportions and the student t-test for comparison of continuous values. Statistical tests were two tailed and p<0.005 was considered significant. Mean Townsend scores were estimated for participants of each survey and separately for responders and non-responders to specific key questions. These questions were on child health status, breast feeding, parental employment, parental and childhood asthma diagnosed by a doctor, household smoking, dampness in the home, childhood wheeze and excess cough.

The level of household socio-economic status was calculated using the Townsend Deprivation Scores derived from respondents postcodes. This used MIMAS software (Manchester Information and Association Services), which converts UK postcodes directly to deprivation scores which range between -6 to -3 (least deprived) and +4 - +12 (most deprived). Townsend scores had been used for the 1991, 1993, and 1998 survey analyses and this socio-economic score was retained for use in the 2006 survey analysis.

The methodological characteristics of the cross-sectional surveys are summarised in Table 1. In the 2006 survey five schools from Wallasey were again included to allow better representation in comparison with the 1991 and 1993 surveys. Other than this sampling difference the methodologies used across the four surveys were identical except for the inclusion of a small number of additional questions on parental education, ADHD, alcohol use, and smoking history in the 1998 and 2006 surveys. The proportion of mothers, or fathers, who completed the questionnaires did not change, although the percentage of questionnaires jointly completed by both parents fell from 15.8% in 1991, to 5.4% in 2006, (p<0.001).

**Table 1:** Methodological characteristics of four cross-sectional surveys

**Characteristics**	**1991**	**1993**	**1998**	**2006**
Number of schools	15	15	10^╫^	15
Pre-notification by letter	Yes	Yes	Yes	Yes
Sampling	Every second child	All children	All children	All children
Re-visits for absenteeism	at 2-4 weeks	at 2-4 weeks	at 2 weeks	at 2 weeks
Inclusion/Exclusion	none	none	none	none
Months of survey	September to December	September to December	September to December	September to December
Age of children	5-11 years	5-11 years	5-11 years	5-11 years
Total number of questionnaires	1872	3746	1964	1074
Mother completed questionnaire	78.5 (1872)	82.8 (3746)	82.0 (1964)	78.0 (1074)
Father completed questionnaire	4.8 (1872)	6.0 (3746)	5.6 (1964)	4.3 (1074)
Both parents completed questionnaire	15.8 (1872)	10.0 (3746)	6.5 (1964)	5.4 (1074)
Questionnaire compliance,%***	92.0% (1872/2035)	87.4 (3746/4288)	78.1 (1964/2514)	30.3 (1074/3540)
Child’s weight	Yes	Yes	Yes	Yes
Child’s height	Yes	Yes	Yes	Yes
Questionnaire length	Reference	unchanged	New questions added*	New questions added**
Incentives	None	None	None	None

*questions on smoking in pregnancy; single parent family.

**Parental level of education, ADHD, alcoholic in pregnancy, grandparent smoking habits during pregnancy

***Number of completed questionnaires/number distributed.

^╫^ Five Wallasey schools excluded.

## Ethical approval

Ethical Committee approval for these surveys was provided by the Royal Liverpool Children’s Hospital NHS Trust, Alder Hey and the Liverpool School of Tropical Medicine Ethical Committees.

## Results

Questionnaire compliance fell from 92% in 1991 to 30.3% in 2006, with the main reduction occurring between 1998 and 2006 (47.8%), (Table 2). There was a substantial variation in questionnaire compliance between individual schools, although the trend to lower compliance in later surveys was consistent across all schools. The lowest school compliance in 2006 was 15.3% for a school which had 98.5% in 1991. Eleven of the 15 schools surveyed in 2006 had compliance below 30%. The highest compliance in 2006 was 55.3%. The Townsend Score for socio-economic deprivation for households of children from the school with highest compliance in 2006 (Liverpool, school number 5, 5.27± 0.25) did not differ from the mean score for households of the school with the lowest compliance (Wallasey, school number 13, 4.94± 0.14, p=0.139).

**Table 2:** Parental questionnaire compliance

	**School Number**	**1991**	**1993**	**1998**	**2006**	**% difference 1991-2006**
Liverpool	1	70/73 (95.9)	309/407 (76.0)	95/150 (63.3)	36/170 (21.2)	74.7
	2	68/86 (79.1)	261/384 (68.0)	171/196 (87.2)	46/200 (23.0)	56.1
	3	137/147 (93.2)	375/403 (93.0)	250/370 (67.5)	70/300 (23.3)	69.1
	4	195/201 (97.0)	201/209 (96.0)	367/400 (91.7)	127/310 (41.0)	56.0
	5	217/232 (93.5)	232/249 (93.0)	359/390 (92.0)	249/450 (55.3)	38.2
	6	83/90 (92.2)	297/323 (92.0)	139/190 (73.1)	51/200 (25.5)	66.7
	7	59/70 (84.3)	450/508 (88.2)	149/211 (71.6)	48/150 (32.0)	52.3
	8	85/86 (98.8)	382/386 (99.0)	129/175 (73.7)	33/185 (17.8)	81.0
	9	173/174 (99.4)	170/175 (97.0)	146/216 (67.5)	83/290 (28.6)	70.8
	10	84/104 (81.7)	130/163 (80.0)	159/216 (73.6)	44/150 (29.3)	52.4
All Liverpool		1171/1263 (92.7)	2807/3207 (87.5)	1964/2514 (78.1)	787/2405 (32.7)	60.0
Wallasey	11	86/92 (94.4)	168/189 (89.0)	-	48/135 (35.6)	58.8
	12	149/159 (93.7)	178/191 (93.0)	-	38/130 (29.2)	64.5
	13	206/209 (98.5)	134/140 (96.0)	-	61/400 (15.3)	83.2
	14	122/151 (80.7)	283/349 (81.1)	-	53/250 (21.2)	59.5
	15	138/161 (85.5)	176/212 (83.0)	-	87/220 (39.5)	46.0
All Wallasey		701/772 (90.8)	939/1081 (86.8)		287/1135 (25.2)	65.6
All Schools		1872/2035 (92.0)	3746/4288 (87.4)	1964/2514 (78.1)	1074/3540 (30.3)	61.7

Brackets=percentage.

*number refers to order of schools visited.

#The same schools were used for both the 1998 and 2006 surveys except for one school which was closed after 1998 and replaced with a proximate school in the 2006 survey.

The percentage of missing responses to the socio-demographic and respiratory health questions are summarised in Table 3 for each of the surveys. The percentage of non-respondents to several specific questions was highest in 2006 (birthweight, child considered healthy or breast fed, smoking and respiratory health questions). Dampness in the home showed an opposite trend with significantly more parents responding to this question in 2006 compared to previous years (p<0.01). The employment questions showed uniform response rates across the four surveys, with consistently more non-responders to the question on paternal, than on maternal employment. The childhood respiratory symptom questions were generally well answered with fewer than 15% non-responders for all surveys.

**Table 3:** Percentage of missing responses to specific questions

**Non -response**	**1991**	**1993**	**1998**	**2006**
Question on sex	12/1872 (0.6)	64/3746 (1.7)	9/1964 (0.4)	0/1074 (0)
Question on age	201/1872 (10.7)	229/3746 (6.1)	186/1964 (9.5)	91/1074 (8.5)
Question on birthweight	78/1872 (4.2)	130/3746 (3.5)	135/1964 (6.9)	258/1074 (24.0)
Child considered healthy	75/1872 (4.0)	99/3746 (2.6)	88/1964 (4.5)	131/1074 (12.2)
Child breast fed	47/1872 (2.5)	97/3746 (2.5)	85/1964 (4.3)	181/1074 (16.9)
Mother employed	96/1872 (5.1)	205/3746 (5.5)	123/1964 (6.3)	88/1074 (8.2)
Father employed	250/1872 (13.4)	700/3746 (18.7)	450/1964 (22.9)	201/1074 (18.7)
Child asthmatic	48/1872 (2.6)	76/3746 (2.0)	71/1964 (3.6)	125/1074 (11.6)
Mother asthmatic	120/1872 (6.4)	239/3746 (6.4)	167/1964 (8.5)	140/1074 (13.0)
Father asthmatic	159/1872 (8.5)	422/3746 (11.3)	323/1964 (16.4)	249/1074 (23.2)
Household smoking	721/1872 (38.5)	1420/3746 (37.9)	40/1964 (2.0)	130/1074 (12.1)
Dampness at home	763/1872 (40.8)	1602/3746 (42.8)	906/1964 (46.1)	206/1074 (19.2)
Wheeze question	124/1872 (6.6)	335/3746 (8.9)	166/1964 (8.5)	118/1074 (10.9)
Cough question	36/1872 (1.9)	134/3746 (3.6)	184/1964 (9.3)	37/1074 (3.5)
Breathlessness question	235/1872 (12.5)	277/3746 (7.4)	116/1964 (5.9)	138/1074 (12.8)

Brackets :percentage

The demographic characteristics of household respondents for the four surveys are summarised in Table 4, which also shows the mean Townsend scores for respondents for each survey. There were significant differences between mean child age, maternal and paternal smoking during pregnancy, and maternal employment between the four surveys (all p<0.01), with reductions in smoking prevalence, and with an increased proportion of mothers in employment in the two most recent surveys. Mean Townsend scores did not differ between respondents across the four surveys. Out-migration estimated by duration of residence in the school area in the previous three years, did not differ between surveys, with approximately three quarters of households resident for at least three years prior to each survey.

**Table 4:** Prevalence of socio-demographic characteristics of household respondents

**Study characteristics**	**1991**	**1993**	**1998**	**2006**	P value*
Mean child age	7.6±1.9(1157)	7.6±(2217)	8.1±1.8(1778)	7.1±2.1(983)	<0.001
Townsend quartiles (Upper quartile +4 to +12)	92.6(1080)	93.3(2174)	92.0(1806)	90.8(657)	0.409
Mean Townsend score	5.50(1080)	5.48(2174)	5.23(1806)	5.26(657)	0.552
Single parent	NA	NA	33.7(1862)	35.9(885)	0.302
Maternal smoking during pregnancy	NA	36.5(3581)	34.8(1874)	28.0(958)	0.004
Paternal smoking during pregnancy	NA	49.6(2926)	45.3(1294)	37.9(763)	<0.001
Household smoking	64.6(1151)	60.0(2326)	58.3(1924)	44.0(944)	<0.001
Mother employed	36.6(1075)	38.3(2229)	49.9(1841)	49.0(986)	0.001
Father employed	66.4(921)	60.6(1913)	73.5(1514)	59.2(873)	0.054
Duration of residence at present address ≥3 years	75.9(1171)	73.1(2368)	72.4(1022)	71.9(888)	0.256
Dampness at home	12.0(1109)	14.0(2144)	15.3(1058)	9.3(868)	0.323

Brackets: sample size.

NA: data not available.

*Analysis of variance.

Mean Townsend scores for responders or non-responders for parents replying to specific core questions are summarised in Table 5. Differences between mean values comparing responders with non-responders were generally small with no consistent differences. Mean Townsend scores of either responders or non-responders to specific questions were marginally higher in 1991 than 2006.

**Table 5:** Townsend scores in responders or non-responders to specific questions

**Question**	**1991**		**1993**		**1998**		**2006**	
	**R**	**NR**	**R**	**NR**	**R**	**NR**	**R**	**NR**
Sex	5.49	7.50	5.47	5.63	5.23	4.70	5.26	NA
Age	5.50	5.85	5.48	5.68	5.23	5.27	5.26	6.33
Birthweight	5.51	5.29	5.47	5.73	5.25	4.90	5.28	5.18
Child considered healthy	5.50	5.62	5.47	5.58	5.22	5.26	5.28	4.91
Child breast fed	5.50	5.35	5.47	6.09	5.25	4.77	5.29	5.18
Mother employed	5.47	5.82	5.47	5.66	5.20	5.60	5.26	5.45
Father employed	5.42	5.80	5.40	5.83	5.12	5.57	5.38	5.74
Child asthmatic	5.50	5.24	5.28	5.25	5.22	4.70	5.22	6.14
Mother asthmatic	5.64	5.73	5.67	5.78	5.23	5.38	5.27	5.09
Father asthmatic	5.65	5.82	5.56	5.75	5.23	5.70	5.16	5.26
Household smoking	5.51	5.15	5.48	5.08	5.23	5.25	5.25	5.36
Dampness at home	5.58	5.43	5.51	5.29	5.36	5.28	5.28	5.24
Wheeze question	5.83	5.75	5.47	5.66	5.22	5.35	5.26	5.20
Excess cough	5.51	4.85	5.47	5.56	5.22	5.54	5.26	5.20
C+W+B+	6.35	6.42	6.04	6.09	5.23	5.07	5.54	5.21

R: responders.

NR: non-responders

CWB: Symptom triad of cough, wheeze and breathlessness.

NA: Not applicable.

## Discussion

It is important to establish the extent to which sampling bias explains survey variation in prevalence of child health outcomes in these surveys. We reported a significant increase in prevalence of maternal asthma with survey year from 1991 to 2006 (6.6% to 13.4%), p<0.001) [3] with a lower childhood prevalence of doctor diagnosed asthma in 2006 than 1998 (29.8% and 19.4%). There was an increasing proportion of hospital admissions between 1991 and 1998 (5.5% to 11.3%, p<0.001), which decreased in 2006 (9.7%). Childhood obesity prevalence was higher in 2006 (14.9%) compared to 1991 (9.2%, p=0.039, linear trend) [5]. Over these periods the pattern of questionnaire compliance showed a linear reduction across all schools with the largest difference between the 1998 and 2006 surveys. The trends were comparable between the ten Liverpool and five Wallasey schools and it is unlikely that non-compliance was a specific problem to Liverpool schools, although these had had an early history of airborne dust exposure from the Liverpool docks. It is likely that the very high survey compliance in 1991 and 1993 was influenced by community concerns about childhood health and dust pollution. The much lower response rate in 2006 is unlikely to be related entirely to amelioration of these environmental exposures which occurred in the interim period.

It is unlikely that questionnaire and sampling variation explained changes in compliance between the four surveys as essentially the same survey instrument, methodology and schools were used for these sequential assessments. The surveys were comparable in terms of questionnaire design and the same core questions were used which comprised the majority of listed questions.

Response rates to general population surveys have been in decline over recent decades, with compliance ranging from 30% to 70% [15,20-27] and the much lower compliance in the 2006 Merseyside survey was consistent with this pattern. This was a major concern, as marked prevalence changes in the survey population were concurrently observed for both childhood and parental asthma [3], and for childhood obesity [5]. The response rates in these school surveys are critical for assessing the validity of the study findings. Response representativeness is more important than the actual response rate in this type of survey [28]. A low response rate of 30% has been proposed as acceptable for patient satisfaction surveys, providing the sample is representative [27]. Supplemental analysis is helpful to confirm that respondents are in fact representative of the population [29]. Ideally analysis of representativeness in relation to non-response bias should compare data from responders who participated in the survey, with non responders who didnot participate in order to measure the potential bias resulting from low response rates [30]. In the present survey this was not possible, and an alternative approach of comparing socio-economic characteristics of responders and non-responders to specific questions was adopted.

Without information on the household characteristics of parents who did not return the questionnaires, it is not possible to determine if these comprised households with different socio-economic profiles. Non-responders have often been shown to differ from responders in terms of a number of socio-demographic and economic variables which are linked to lifestyles, attitudes and beliefs [24,31]. The pattern of non-response bias is difficult to assess because characteristics of those who are contacted, but refuse to participate, will not be available [32]. There were no mean differences in socio-economic deprivation indices between respondents across the four surveys. This population was predominantly Caucasian (>94%) [17] throughout these survey years, and there were no major shifts in ethnic composition, indicating there were no substantial ethnic demographic changes.

The pattern of parental non-responses to specific questions varied across the four surveys indicating some influence of survey year on response characteristics. Non-responses to some core questions were higher in 2006, when survey compliance was also lowest, indicating a greater reluctance to provide answers to specific questions in the 2006 survey. This was not the case for the response to the question on dampness in the home, for which the response rate had improved in 2006, concurrent with social improvements in housing in the later survey years. A significant change in the 2006 survey was the higher proportion of employed mothers. As mothers were mostly responsible for completing questionnaires, a busier working lifestyle might explain increased reluctance to respond to time consuming school-questionnaires. The estimated time for completing the school questionnaire was approximately 20 minutes. The proportion of single parent households, and the mean Townsend scores, did not vary between surveys. Similarly, duration of residence at the present address for more than three years did not significantly differ across surveys. This suggests that despite a reduction in compliance in 2006, there were no major changes in household socio-demographic profiles between surveys for parents who returned questionnaires. This is re-assuring in terms of response bias related to social deprivation. Examining this in further detail in relation to mean Townsend scores for responders or non-responders to specific questions, showed scores were slightly lower in 2006 than 1991, but this occurred for both these groups. There was no pattern towards lower scores for households with non-responses to specific core questions.

## Study limitations

As characteristics of questionnaire non-respondents were not available it was not possible to have a comparative group of parents who refused to participate. Record linkage to other data sources would be an alternative method to obtain information on non-respondents [30], but ethical approval for this was not available for these surveys.

Without information on the household characteristics of parents who did not return the questionnaires, it is not possible to determine if these comprised households with different socio-economic profiles. Non-responders have often been shown to differ from responders in terms of a number of socio-demographic and economic variables which are linked to lifestyles, attitudes and beliefs [24,31]. The pattern of non-response bias is difficult to assess because characteristics of those who are contacted, but refuse to participate will not be available [32].

## Conclusions

These surveys provide the first evidence using a standardised community survey methodology for child health assessment of a major shift in parental questionnaire compliance for an urban population over a 15 year period to 2006. It is unlikely that the methodological differences between surveys explained changes in compliance as the same survey instrument, methodology and schools were used for all the sequential surveys. The fall in response rates to school surveys over the past several years has occurred at the same time as a number of important changes in the compulsory education system in United Kingdom (UK) [33]. The decreased parental compliance may relate to increasing frequency of questionnaire surveys occurring in schools related to various audits, monitoring and evaluation activities, lack of available time for employed parents, the length of questionnaires. Minor changes in demographic characteristics of the surveyed sample, and changing attitudes of respondents to sensitive questions may be contributory. Heightened concern about airborne dust pollution between 1991 and 1993 was an important stimulus to questionnaire compliance at that time, and the removal of these exposures may have reduced community interest in health assessments. The present analysis provides evidence for response representativeness over the survey periods, despite the parallel changes in compliance.

Parental compliance is an emerging major problem for cross-sectional surveys of childhood health in urban areas. This limitation must be considered in community survey designs and requires better capture of socio-demographic data on non-respondents, and inclusion of in built methods which allow for assessment of response representativeness.

## Competing interests

The authors declare that they have no competing interests.

## Authors' contributions

G.K and B.B contributed equally to the paper and read and approved the final manuscript.
